# Genomic sequencing has a high diagnostic yield in children with congenital anomalies of the heart and urinary system

**DOI:** 10.3389/fped.2023.1157630

**Published:** 2023-03-14

**Authors:** Erika T. Allred, Elliot A. Perens, Nicole G. Coufal, Erica Sanford Kobayashi, Stephen F. Kingsmore, David P. Dimmock

**Affiliations:** ^1^ Department of Pediatrics, University of California, San Diego, CA, United States; ^2^ Rady Children's Institute for Genomic Medicine, San Diego, CA, United States; ^3^ Department of Pediatrics, Children's Hospital of Orange County, Orange, CA, United States

**Keywords:** genomics, congenital anomalies, kidneys, heart, sequencing, hospital

## Abstract

**Background:**

Congenital heart defects (CHD) and congenital anomalies of the kidney and urinary tract (CAKUT) account for significant morbidity and mortality in childhood. Dozens of monogenic causes of anomalies in each organ system have been identified. However, even though 30% of CHD patients also have a CAKUT and both organs arise from the lateral mesoderm, there is sparse overlap of the genes implicated in the congenital anomalies for these organ systems. We sought to determine whether patients with both CAKUT and CHD have a monogenic etiology, with the long-term goal of guiding future diagnostic work up and improving outcomes.

**Methods:**

Retrospective review of electronic medical records (EMR), identifying patients admitted to Rady Children's Hospital between January 2015 and July 2020 with both CAKUT and CHD who underwent either whole exome sequencing (WES) or whole genome sequencing (WGS). Data collected included demographics, presenting phenotype, genetic results, and mother's pregnancy history. WGS data was reanalyzed with a specific focus on the CAKUT and CHD phenotype. Genetic results were reviewed to identify causative, candidate, and novel genes for the CAKUT and CHD phenotype. Associated additional structural malformations were identified and categorized.

**Results:**

Thirty-two patients were identified. Eight patients had causative variants for the CAKUT/CHD phenotype, three patients had candidate variants, and three patients had potential novel variants. Five patients had variants in genes not associated with the CAKUT/CHD phenotype, and 13 patients had no variant identified. Of these, eight patients were identified as having possible alternative causes for their CHD/CAKUT phenotype. Eighty-eight percent of all CAKUT/CHD patients had at least one additional organ system with a structural malformation.

**Conclusions:**

Overall, our study demonstrated a high rate of monogenic etiologies in hospitalized patients with both CHD and CAKUT, with a diagnostic rate of 44%. Thus, physicians should have a high suspicion for genetic disease in this population. Together, these data provide valuable information on how to approach acutely ill patients with CAKUT and CHD, including guiding diagnostic work up for associated phenotypes, as well as novel insights into the genetics of CAKUT and CHD overlap syndromes in hospitalized children.

## Introduction

Congenital birth defects frequently have an underlying molecular etiology. For example, there are over 40 genes each that are associated with congenital anomalies of the kidney and urinary tract (CAKUT) and congenital heart defects (CHD) (1, 2). Causative molecular variants that span these two systems are less well characterized, and we sought to better define these.

CAKUT represent a heterogeneous group of disorders ranging from mild urinary tract dilation to bilateral renal agenesis (1). Kidney malformations are one of the most common congenital anomalies, representing 20%–30% of all prenatally detected anomalies (3, 4). CAKUT are known to carry significant morbidity in the pediatric population, accounting for 45% of chronic kidney disease (CKD) and about 30% of end stage kidney disease (ESKD) (5). Over 40 single-gene defects have been implicated in CAKUT, with a Mendelian molecular etiology detected in up to 26% of all cases (1, 6). It is estimated that hundreds of additional monogenic defects have yet to be discovered in CAKUT, with identification complicated by incomplete penetrance and variable expressivity, along with suspected contributions from epigenetic and environmental factors (6, 7).

Additional causes of CAKUT include many well-known genetic syndromes which present with extra-renal manifestations, such as Fraser syndrome, CHARGE syndrome, and DiGeorge (8). Interestingly, each of these syndromes include CHD in their phenotypes. CHD is the primary cause of congenital anomalies in children, representing almost one third of all congenital anomalies, and is the most common cause of death before the age of one year (9, 10). CHD also has a strong genetic basis, with over 40 monogenic gene defects identified (2).

Several studies have highlighted a strong clinical correlation between non-syndromic CHD and CAKUT, with 20%–30% of all patients with CHD also found to have CAKUT (8). These findings suggest a genetic overlap between the etiology of both anomalies, which has been supported by several mouse models demonstrating a single gene to be responsible for both CHD and CAKUT (7). The monogenic overlap between CHD and CAKUT has yet to be fully evaluated in human studies, with only sporadic case reports describing single gene causes of both (11, 12). In addition, the current known monogenic causes of CAKUT do not overlap with those of CHD, despite the well-known clinical overlap and similar embryologic origin of cardiac and kidney development (13, 14). However, there are no human studies specifically evaluating the genetics of individuals with both CAKUT and CHD, and thus the contribution of monogenic defects to this population is still unknown.

There is a significant clinical need to further identify and characterize the genetic underpinnings and presenting phenotypes of patients with both CAKUT and CHD due to the well-known interdependent relationship of the two organ systems. Cardiorenal syndrome encompasses a spectrum of disorders in which dysfunction of one organ system induces dysfunction in the other (15). This syndrome is a significant cause of morbidity and mortality in hospitalized patients, with both the Studies of Left Ventricular Dysfunction (SOLVD) Prevention Trial and Candesartan in Heart Failure: Assessment of Reduction in Mortality and Morbidity (CHARM) study noting almost a doubling in the mortality rate of heart failure patients if they experienced a decline in kidney function (16, 17). This impact is magnified and more likely to occur in patients who already possess a congenital anomaly in either organ system.

Due to the pervasiveness and significant disease burden of CAKUT and CHD in the pediatric population, we performed a descriptive and analytic cross-sectional evaluation of the prevalence of patients with both CAKUT and CHD. We analyzed patients at a single-center tertiary hospital who had diagnostic whole exome sequencing (WES) and/or whole genome sequencing (WGS). Using this information, we sought to further elucidate the phenotypic spectrum and candidate genetic underpinnings of patients affected by both CAKUT and CHD. We anticipate that this information will improve patient outcomes by increasing providers' diagnostic and prognostic capabilities, with future aspirations to include implementation of prophylactic measures and the reduction of unnecessary interventions.

## Methods

### Study design

We performed a retrospective chart review of electronic medical records (EMR) of all patients who had received diagnostic WES and/or WGS and had ICD10 diagnosis codes of Q20–28 (congenital malformations of cardiac chambers and connections) or Q60–69 (included congenital malformations of the urinary system) that were cared for at Rady Children's Hospital between January 2015 and July 2020. Available individual patient imaging studies were then evaluated to identify patients with both CAKUT and CHD. Imaging studies reviewed included any or all of the following: kidney and bladder ultrasounds (RUS), vesicoureteral cystograms (VCUG), dimercaptosuccinic acid (DMSA) scans, mercaptoacetyltriglycine (MAG3) scans, computed tomography (CT) scans of the abdomen, magnetic resonance imaging (MRI) of the abdomen, CT scans of the heart, MRIs of the heart, and echocardiograms (ECHO). Patients were excluded if their imaging failed to show *both* CAKUT and CHD, if the EMR represented the parent of a child under investigation, if cardiac imaging revealed only a patent foramen ovale (PFO) or patent ductus arteriosus (PDA) that resolved by 12 months of age without surgical intervention, or if kidney imaging was only notable for hydronephrosis that resolved within the first year of life without intervention. Data collected included: demographic data (e.g., sex, race/ethnicity, date of birth), diagnoses, patient age at time of WGS/WES sequencing, additional imaging, presenting phenotype, maternal pregnancy history, human phenotype ontology (HPO) terms, and all genome wide sequencing (GWS) results.

This study is limited due to its analysis of a single center with a small population. In addition, results were interpreted with the recognition that genetic testing was confined to WGS and WES only. These limitations will be explored further in the discussion section of this paper.

### Diagnostic WGS and WES analysis and interpretation

Clinical WES and WGS were performed in laboratories accredited by the College of American Pathologists and certified through the Clinical Laboratory Improvement Amendments. The analysis and interpretation protocol were adapted from the NSIGHT2 study (18).

WGS results were reinterpreted for this study using Opal Clinical (Fabric Genomics). Genes associated with CAKUT and CHD were identified using the Phenolyzer algorithm (19). Opal then re-annotated variants using the resultant gene panel for the Variant Annotation, Analysis & Search Tool (VAAST) variant prioritizer Phevor algorithm, re-ranking with the specific Human Phenotype Ontology (HPO) terms, “abnormal heart morphology” and “abnormal renal morphology”. Automatically generated, ranked results were manually re-interpreted through iterative Opal searches and filters.

## Results

**Figure 1:**
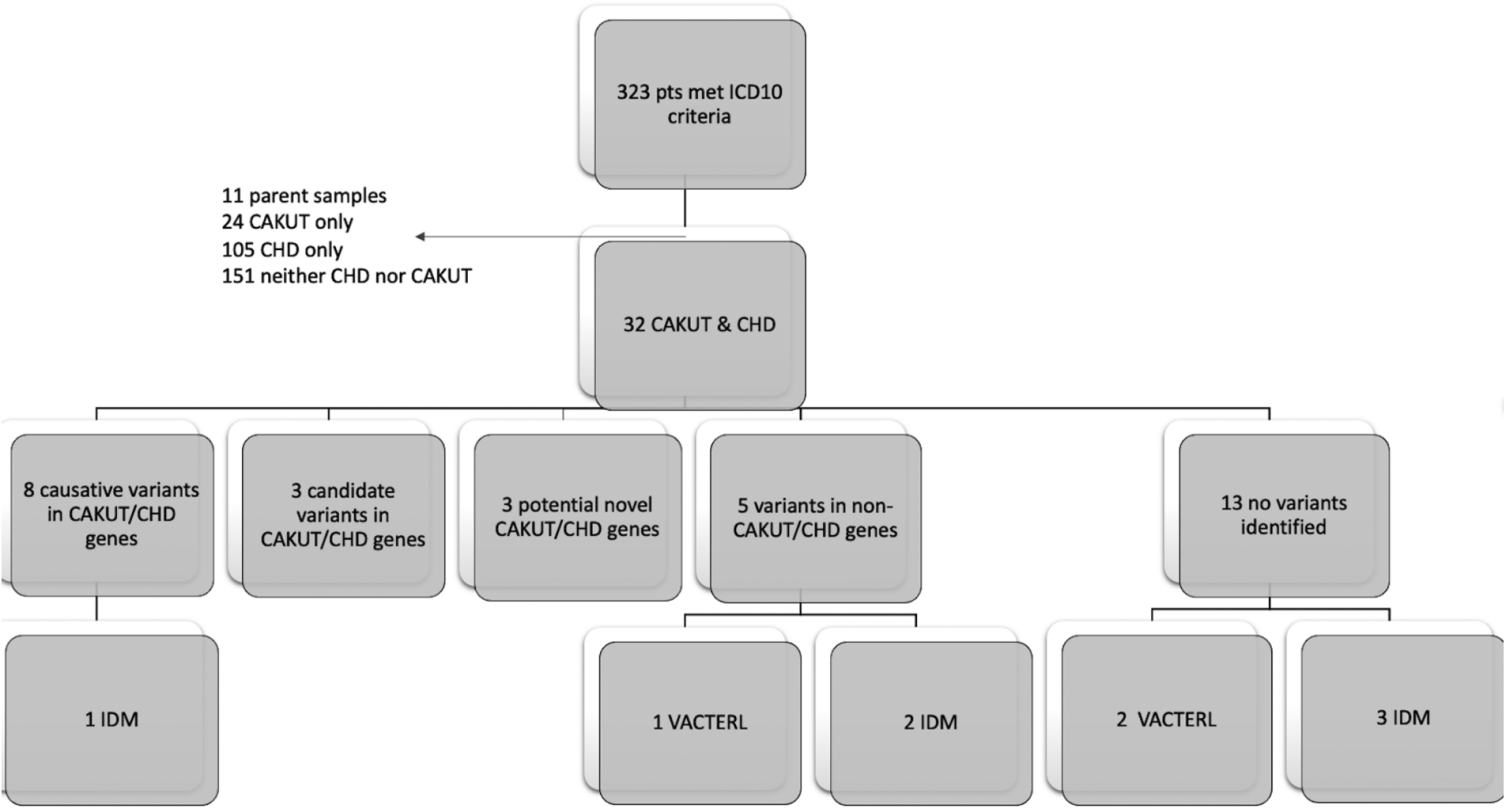
Flow diagram of patient inclusion and results: Infant of diabetic mother (IDM), Congenital heart defect (CHD), Congenital anomaly of kidney and urinary tract system (CAKUT).

We identified 323 cases with the defined ICD10 diagnosis codes corresponding to congenital malformations of cardiac chambers and connections or the urinary system and diagnostic WGS or WES results (Figure 1). Of those cases, 105 were excluded after being identified as isolated CHD, 24 were excluded for isolated CAKUT, 11 were parental charts of children under investigation, and 105 had neither CAKUT nor CHD as defined in the current study. Twenty-three percent of all cases with CHD were also found to have CAKUT. In total, we identified 32 cases with CAKUT and CHD who had received diagnostic WGS or WES. Demographic information is detailed in Table 1. Fourteen cases underwent WES and 18 cases underwent WGS. Due to the limited number of overall cases, WGS and WES results were interpreted as one cohort. We identified eight cases with pathogenic or likely pathogenic variants in genes known to cause CAKUT and CHD. Three cases had four variants of uncertain significance (VUS) suspected to be disease causing in genes known to cause CAKUT and CHD. Three cases had four variants (2 classified as pathogenic, 2 VUS) in genes not previously associated with CAKUT and CHD. Five cases had eight reported variants (some cases had more than one variant) in genes not previously associated with CAKUT and CHD and not suspected to be the cause of the CAKUT/CHD phenotype due to a lack of supporting literature. Thirteen cases had no variants identified *via* focused reinterpretation of WGS or WES for genes and disorders associated with CAKUT or CHD. Of the 18 cases without genetic results related to their CAKUT/CHD phenotype, four were infants of diabetic mothers (IDM), three patients were found to have VACTERL association, and one case had both a diabetic mother and VACTERL association. Therefore, eight out of the 18 cases without a causative molecular diagnosis (44%) were suspected of having an identifiable, putative etiology for their CAKUT/CHD phenotype. All causative, candidate, and novel variants were found in genes associated with genetic syndromes, with the exception of *PLD1,* which is associated with CHD alone (Developmental cardiac valvular defect, MIM 212093). Please see Table 2 for a detailed evaluation of all identified variants.

**Table 1:** Study population characteristics.

	Total (*N*, %)	CAKUT/CHD-Related Molecular Diagnosis
Total number of patients	32	14 (44)
Males	18 (56)	5 (28)
Females	14 (44)	9 (64)
Genomes	18 (56)	8 (39)
Exomes	14 (44)	6 (43)
Median age at time of testing, years	3	3
Race*:		
Asian OR Pacific Islander OR Hawaiian	5 (15)	2 (40)
Asian AND Black	1 (3)	1 (100)
Black/African American	1 (3)	1 (100)
Black/African American AND White	1 (3)	1 (100)
Hispanic/Latinx	3 (9)	2 (67)
Other	1 (3)	0 (0)
White	20 (62)	7 (35)

* Race was self-reported.

**Table 2:** Identified variants *via* WGS/WES.

Gene	MIM Gene ID#	MIM Phenotype ID#	Chromosome: genomic coordinates	Size	Variant	Zygosity (Inheritance)	Classification
**Causative Variants**							
*Patient 1007*							
*CHD7*	608,892	214,800	8:61,654,796		c.807del *p*.Ala270ProfsTer35	Heterozygous (not maternal)	Pathogenic
*Patient 1008*							
*KAT6B*	605,880	606,170	10:76,788,744	* *	c.4162C > T *p*.Gln1388Ter	Heterozygous (*de novo*)	Likely pathogenic
35 Genes		614,671		847KB	Chr16:29,453,701-30,300,900, dup (16p11.2)	Heterozygous (paternal)	Pathogenic
*Patient 1010*							
*FGF10*	602,115	149,730		171KB	Chr5:44,247,363-44,418,819, del(5p12)	Deletion (paternal)	Pathogenic
*Patient 1011*							
*GREB1l*	*617,782*	617,805	18:19,076,462		c.3194C > T, *p*.Thr1065ile	Heterozygous (maternal)	Likely pathogenic
*Patient 1002*							
*ADNP*	611,386	615,873			c.2157C > G, *p*.Tyr719Ter	Heterozygous (De Novo)	Pathogenic
*Patient 1003*							
*NSD1*	606,681	117,550			c.4039delA, *p*.Arg1347GlyfsX25	Heterozygous (De Novo)	Pathogenic
*Patient 1005*							
*EFTUD2*	603,892	610,536			c.1861-2A > C, IVS18-2A > C	Heterozgyous (De Novo)	Pathogenic
*Patient 1009*							
280 Genes		190,685		48,130KB	Chr21:1-48,129,895, dup (21p13q22.3)	Duplication (De Novo)	Pathogenic
**Candidate Variants**							
*Patient 2007*							
*ACTA2*	102,620	611,788	10:90,701,120		c.482 T > G *p*.Val161Ala	Heterozygous (maternal)	VUS
*Patient 2001*							
*ATP6AP2*	300,556	300,423			C.212G > A, *p*.Arg71His	Heterozygous (De Novo)	VUS
*Patient 3013*							
*PLD1*	602,382	212,093			c.55G > A, *p*.Ala19Thr	Heterozygous (maternal)	VUS
*PLD1*	602,382	212,093			c.1504C > T, *p*.Arg502Trp	Heterozgyous (paternal)	VUS
**Novel Variants**							
*Patient 2006*							
*LRP4*	604,270	212,780	11:46,895,119		c.4255A > G *p*.Met1419Val	Heterozygous (paternal)	VUS
*LRP4*	604,270	212,780	11:46,900,844		c.2837C > G *p*.Pro946Arg	Heterozygous (maternal)	VUS
*Patient 1004*							
*PCDH15*	605,514	601,067			c.733C > T, *p*.Arg245Ter	Heterozygous (maternal)	Pathogenic
*Patient 1006*							
*VPS4A*	609,982	619,273			c.298G > A, *p*.Glu100Lys	Heterozygous (*de novo*)	Likely Pathogenic
**Non-CAKUT/CHD Variants**							
*Patient 2005*							
*KRT25*	616,646	616,760	17:38,911,310		c.214C > T *p*.Arg72Trp	Hemizygous (maternal)	VUS
*KRT25*	616,646	616,760		6KB	Chr17:38,909,549-38,915,466, del (17q21.2)	Heterozygous (paternal)	VUS
*Patient 1001*							
*FLG*	135,940				c.3892delT, *P*.Ser1298LeufsX148	Heterozygous (maternal)	Pathogenic
*PKD2*	173,910				c.2291A > T, *p*.Gln764Leu	Heterozygous (maternal)	VUS
*Patient 2002*							
*GCK*	138,079				C.137G > T, *p*.Arg46Met	Heterozygous (maternal)	VUS
*POLG2*	604,983				c.341G > A, *p*.Trp114Ter	Heterozygous (paternal)	VUS
*Patient 2004*							
*OBSL1*	610,991	612,921			c.5108G > A, *p*.gly1703Glu	Heterozygous (paternal)	VUS
*OBSL1*	610,991	612,921			c.1255C > T, *p*.Arg419Cys	Heterozygous (maternal)	VUS
*Patient 2003*							
*MT-TK*	590,060				m.8340G > A	Heteroplasmy 2% (maternal)	Pathogenic
*MT-TS2*	590,085				m.12223A > G	Homplasmic	VUS

Novel, putative CAKUT/CHD gene associations were substantiated by evidence demonstrating presence of the protein product in both organ systems or research supporting physiologic or structural abnormalities in one organ system but not the other (Table 3). For example, *VPS4A* has been associated with cerebellar hypoplasia, cataracts, impaired intellectual development, congenital microcephaly, dystonia, dyserythropoietic anemia, and growth retardation (CIMDAG syndrome, MIM 619273) and with recurrent urinary tract infections, which frequently indicates an underlying structural anomaly (e.g., vesicoureteral reflux or obstruction) (19). However, there were no reports of CHD associated with this disorder.

**Table 3:** Potential novel CAKUT/CHD genes.

Gene	Variants	Syndrome	Prior Literature
***PCDH15***	c.733C > T *p*.Arg245Ter	Usher Syndrome	PCDH15 -CD2 expression has been found in heart, kidney, thymus, spleen, testis, retina and cochlea.
***VPS4A***	c.298G > A *p*.Glu100Lys	CIMDAG Syndrome	Found in individuals with frequent UTIs. No reported CHD.
***LRP4***	1. c.4255A > G *p*.Met1419Val 2. c.2837C > G *p*.Pro946Arg	Cenani-Lenz Syndactyly Syndrome	Causes renal agenesis. No reported CHD

In total, GWS identified a suspected genetic etiology in 44% of cases with CAKUT/CHD phenotypes, with another 13% identified as having a likely environmental etiology, and an additional 13% with VACTERL syndrome. In total, 70% of cases were determined to have a suspected etiology for their presentation.

The most common CAKUT phenotype identified was hydronephrosis, seen in 22/32 (69%) of all cases. The most common CHD phenotype identified was septal defects, identified in 22/32 (69%) of all cases. Additional phenotypic information can be found in Table 4.

**Table 4:** CAKUT and CHD phenotype*.

	*N*	%
**CAKUT Phenotype:**		
Hydronephrosis	22	69
Vesicoureteral reflux	5	16
Renal Agenesis	3	9
Hypo/Dysplasia	3	9
Ectopy	1	3
**CHD Phenotype:**		
Sepal Defects	22	69
Valvular Anomalies	9	28
Dilated Heart Chambers	5	16
Coarctation of Aorta	4	13
Tetralogy of Fallot	2	6
Hypoplastic Left Heart	1	3
Transposition of Great Arteries	1	3

*Patients may have more than 1 phenotype in each category.

We also identified additional structural anomalies in other organ systems in 28 out of the 32 cases (88%). Additional organ systems were classified based on ICD10 codes and included: respiratory, eye/ear/face/neck, cleft lip and palate, nervous system, musculoskeletal (msk), digestive, genital, and other circulatory. The most common additional organ systems affected were the nervous system (53% of cases) and musculoskeletal systems (44% of cases). Structural brain anomalies were the most common anomalies identified in the nervous system. Pectus anomalies and scoliosis were the most common anomalies identified in the musculoskeletal system. Additional organ system involvement is detailed in Table 5.

**Table 5:** Additional organ systems with structural congenital anomalies.

Pt ID	Gene	Respiratory	Eye, ear, face, neck	Cleft lip & palate	Nervous System	MSK	Digestive	Genital	Other Circulatory
1007	*CHD7*	X	X						
1005	*EFTUD2*		X	X	X	X			
1008	*KAT6B*		X		X	X	X		
1002	*ADNP*	X			X	X			
1010	*FGF10*	X							
1003	*NSD1*					X			
1011	*GREB1L*				X				
2007	*ACTA2*	X							
2001	*ATP6AP2*				X	X		X	
1004	*PCHD15*	X	X		X	X			
1006	*VPS4A*				X				
2006	*LRP4*				X				
3011			X		X	X			
3012					X	X	X		
2003					X	X			
3004					X		X	X	
2002	*GCK POLG2*					X			
3014					X			X	
2005	*KRT25*				X				
2004	*OSLB1*				X	X			
1001	*FLG*					X		X	
3006		X							X
3001					X	X			X
3002						X		X	
3003					X				
3008					X				
3015				X			X		
3007								X	
		6/32 (19%)	5/32 (16%)	2/32 (6%)	17/32 (53%)	14/32 (44%)	4/32 (13%)	6/32 (19%)	2/32 (6%)

Patients with only CAKUT and CHD and no other organ system involvement included the following case ID numbers: 3005, 3010, 1009, and 3013. Two of these cases did not have an identifiable etiology for their CAKUT and CHD phenotype. ID 1009 was found to have trisomy 21 and ID 3,013 had two suspected disease-causing variants in the PLD1 gene, the only gene in this group without an associated syndromic phenotype.

## Discussion

### Rare genes identified

In this study of a small, but ancestrally diverse cohort of 32 cases diagnosed with both CAKUT and CHD, we identified molecular etiologies in 14 cases (44%). This yield is higher than that observed for patients with either CAKUT or CHD alone, suggesting that a genetic etiology should be highly suspected and evaluated for in patients with both CAKUT and CHD. We identified 12 distinct monogenic disorders, representing 86% of all genetic diagnoses (Table 2). This yield is significantly higher than either CHD or CAKUT alone, for which most identified genetic causes are due to copy number variants (CNVs) or chromosomal abnormalities (13, 14). Recently, however, a paper published by Sweeney et al. in 2021 reports point mutations as the most common genetic etiology for a small cohort of CHD cases with WGS (20). It is feasible that the differences in genetic defects identified in our study and Sweeney et al. vs. historical literature are due to the technologic limits of genetic testing prior to the advent of WES and WGS. With increasing use of more comprehensive diagnostic technology, it is likely our identification of common genetic defects will continue to expand. These results suggest that monogenic causes of both CAKUT and CHD are much more common than previously expected and may differ in genetic etiology from CHD and CAKUT alone. In addition, these results suggest that genetic testing by GWS should be considered more frequently in the diagnostic evaluation of children with CHD, CAKUT, or both of unknown etiology.

Our study identified 15 monogenic causes of both CAKUT and CHD that would not have been identified by standard gene panels for CHD or CAKUT alone. Of the four common gene panels used for etiologic assessment of CAKUT, only 1–2 of our 14 patients (7%–14%) would have received a diagnosis, depending on the panel used. Genes identified in our patient population and included on CAKUT gene panels were *GREB1l* and *LRP4.* CHD gene panels were more likely to identify a molecular etiology, with most expanded panels ( > 50 genes) expected to yield a diagnosis in 2–5 of our 14 patients (14%–36%), depending on the panel used. CHD-associated genes identified in our patient population and included in gene panels included: *ACTA2, CHD7, EFTUD2, NSD1* and *PLD1*. Overall, these findings demonstrate substantial genetic heterogeneity in this population, and importantly they suggest a lack of significant overlap with genes commonly identified in isolated cases of CHD or CAKUT. Our results also suggest that these patients are likely to be underdiagnosed by gene panels, often the first genetic test utilized. We suggest that the etiologic evaluation of children with both CAKUT and CHD should be with diagnostic GWS and not by gene panel sequencing. The diagnostic superiority of GWS over gene panels is likely as a result of unrecognized phenotype expansion.

### Novel CAKUT and CHD genes

We identified three putative novel gene associations with CAKUT and CHD herein (*LRP4, PCDH15, VPS4A*). These genes contained a suspected disease-causing variant in an affected child, but the gene had not been previously associated with CAKUT and CHD. In one such case, variants in the *PCDH15* gene have been associated with Usher syndrome (MIM 601067), which presents with hearing and vision loss, and matched the clinical phenotype for this patient (21). The protein product of *PCDH15* is also expressed in other organ systems, including the heart and kidney (21). Thus, it was feasible that deleterious variation in this gene could be associated with both CAKUT and CHD phenotypes, as observed in this case. The variant identified in our patient was predicted to cause loss of function of the protein product through truncation or nonsense-mediated mRNA decay. Additional studies will be required to confirm that this is a recurrent phenotype expansion.

The other two genes identified have been implicated in kidney anomalies but have no prior reports of association with CHD (22, 23). The *LRP4* gene has been associated with renal agenesis (23). Of the two variants identified in our patient, in silico prediction tools supported a deleterious effect of one variant on the protein structure/function due to the alteration of a highly conserved amino acid, and discordant results for the other variant. The *VPS4* gene is associated with recurrent kidney infections, generally suggesting an underlying kidney anomaly (22). In addition, the GeneDx in silico analysis supported a deleterious effect of the VPS4A missense variant on protein structure/function. Additional phenotype expansion case reports or *in vivo* studies will be required for causal gene association with CHD and CAKUT.

### Environmental factors

Several environmental teratogens have also been associated with CAKUT and CHD anomalies including certain drugs, chemicals, and fetal exposure to hyperglycemia (24). Fetal structural defects are three to four-fold higher in infants born to diabetic mothers. These anomalies can affect a number of organ systems including: neurologic, gastrointestinal, kidney, and heart (25). Our study identified six cases with mothers who had diabetes requiring insulin therapy during pregnancy. Of note, five of these cases (83%) did not have an identifiable genetic etiology for their presentation. These findings suggest that genetic testing may have a low yield in patients with a likely environmental etiology for CAKUT and CHD.

Our study also identified three cases with VACTERL association and no known genetic etiology. The etiopathogenesis of VACTERL association is still unknown, but theories include possible teratogenic exposures, a malformation cascade, combination of environmental and epigenetic factors, or disturbances in developmental processes essential to all affected organ systems (24, 26). Overall, of the 18 cases in our study without a genetic diagnosis, seven cases (39%) had a suspected environmental etiology for their presentation. Our study suggests that the majority of patients with CAKUT and CHD will have an identifiable etiology for their anomalies, and thus a thorough history should evaluate for exposure to environmental teratogens, physical exam and imagining should evaluate for VACTERL association, and genetic evaluation should include WES or WGS.

### Expanding the phenotype of patients with CAKUT and CHD

Previously CAKUT and CHD have both been found as solitary anomalies or co-existing with other organ system malformations (3, 4, 7, 8, 27–31). Overall, 40%–60% of patients with a CAKUT or CHD have an additional extra-renal or extra-cardiac abnormality (7, 8, 27–30). CHD and CAKUT are known to co-occur in 25%–30% of affected individuals, more than any other concurrent organ system anomalies (7, 8, 31). During study subject ascertainment, 23% of hospitalized CHD cases were found to also have an identifiable CAKUT, in accord with published literature (7, 8, 31). Electronic medical record review also identified additional organ system anomalies in 28 cases (88%) with CAKUT and CHD (Table 5). The most commonly identified extra-renal/cardiac malformations were those in the nervous system (53% of all cases), with mainly brain malformations, and musculoskeletal system (44% of all cases), with primarily scoliosis and pectus anomalies identified. While underpowered for statistical analysis, certain organ system associations appeared to co-occur more commonly in those with genetic diagnoses or those without. For example, respiratory and eye/ear/face/neck anomalies were more likely to occur in those patients with a genetic etiology than those without, with nine out of 11 malformations (82%) identified in those with an underlying genetic diagnosis. In contrast, cleft lip/palate, musculoskeletal, and nervous system anomalies occurred at about an equal rate in cases with and without a genetic diagnosis. Digestive, other circulatory, and genital anomalies occurred more frequently in cases without a molecular diagnosis, with 10 out of 12 malformations (83%) identified in those without a genetic etiology. If substantiated in larger cohorts, these findings may guide prioritization for diagnostic GWS.

Overall, we found significantly more extra-renal/cardiac malformations in our patients with both CAKUT and CHD than has been reported with either CAKUT or CHD alone (7, 8, 31). These findings may be related to the high rate of genetic syndromes identified in this study, with 13 of our 14 genetic diagnoses (93%) herein related to a previously identified syndrome. However, additional structural malformations were identified just as commonly in those with environmental causes and no identifiable etiology for their presentation. Patients with CHD or CAKUT and additional organ system anomalies have higher morbidity and mortality, and thus our data may be biased, since hospital admission was among the selection criteria (27, 29). Despite this possible skewing, these results still provide valuable information on what additional diagnostic work up should be considered, and suggest that hospitalized pediatric patients with both CAKUT and CHD should be evaluated for additional organ system malformations, most specifically in the musculoskeletal and nervous systems.

### Limitations

Our study has several limitations that need to be addressed. Primarily, this is a small study at a single tertiary center, which limited our ability to adequately power this analysis and provide any statistical evaluations. Secondly, we evaluated only hospitalized patients with CAKUT and CHD. This filter was set specifically to evaluate the sickest of this population, in order to better direct acute care and management. However, in limiting our population in this fashion, our findings may not be generalizable to patients who have not been hospitalized.

In addition,, we only evaluated those patients with exome and genome sequencing, missing those in which a genetic diagnosis is made *via* microarray, karyotype, or gene panels only. Without this information, we do not know if the patients diagnosed through these testing strategies would present with a different phenotype than our current patient population. It is also unclear if some of the more commonly known CAKUT/CHD genes were screened out with these testing methodologies, thus negating the need for more thorough genetic testing strategies. As reanalysis was only applied to those with WGS, it is also possible that additional genetic diagnoses were missed in those patients who only had WES.

Furthermore, we recognize the limitations of WES in comparison with WGS. Due to the nature of WES, this type of sequencing may have missed small CNVs, mitochondrial variants, intronic variation, repeat expansion, mobile insertion elements and coverage of PCR-free genomes (32). Bertoli-Avella et al. found a 14% increase in diagnostic yield of WGS over WES in a cohort of over 300 patients (32). Taking these findings into account, it's possible we may have missed up to 2 molecular diagnoses in our WES cohort, that may have been identified had WGS been available.

Our study did not identify any repeat implicated genes in our population. These findings likely signify a lack of saturation due to our small patient cohort.

### Future directions

Although our study evaluated a small subset of patients at a single center, it is the first of its kind to specifically evaluate the genotype and phenotype of this patient population. This study provides a launching point to encourage additional studies interrogating all genetic testing in this patient population and to expand the evaluation to patients in both the inpatient and outpatient setting across multiple centers. Additional evaluation should also focus on those patients with CAKUT and CHD and no other affected organ system, as these may have a different and more enriched diagnostic yield.

## Data Availability

The data analyzed in this study is subject to the following licenses/restrictions: All data associated with this study are present in the paper. While no DNA sequence was generated as a part of this published work, all novel DNA sequence variants have been uploaded to ClinVar under our institutional identifier, Organization ID: 506081. Requests to access these datasets should be directed to etallred@health.ucsd.edu.
